# Does Trauma Center Volume Matter? An Analysis of Trauma Center Volume on Outcome Using the TQIP/NTDB Database

**DOI:** 10.3390/jcm13226655

**Published:** 2024-11-06

**Authors:** Alan Cook, Nicholas J. Larson, Heidi M. Altamirano, Brittany Ray, Brandi Pero, Musharaf Mohiuddin, Rebecca Swindall, Carly Wadle, David J. Dries, Benoit Blondeau, Frederick Rogers

**Affiliations:** 1 Department of Surgery, Health Science Center at Tyler, University of Texas, UT Health East Texas, 11937 U.S. Hwy. 271, Tyler, TX 75708, USA; adcookmd@gmail.com (A.C.); musharaf.mohiuddin@uttyler.edu (M.M.); rebecca.swindall@uttyler.edu (R.S.); carly.wadle@uttyler.edu (C.W.); 2 Department of Surgery, Regions Hospital, 640 Jackson Street, Saint Paul, MN 55101, USA; nicholas.j.larson@healthpartners.com (N.J.L.);

**Keywords:** trauma, trauma centers, trauma systems, admission volume, trauma center verification

## Abstract

**Background:** Increasing trauma center admission volume is said to decrease mortality. Evidence supporting this position is dependent upon patient groups and the time period studied, and gaps remain. We evaluated the effect of annual volume of critically injured patients on hospital mortality, comparing two time periods. The effect of critically injured patient volume on risk-adjusted mortality was hypothesized to decrease over time. **Methods:** This was a retrospective cohort study comparing data from an early group (2007–2011) and late group (2017–2021) of the National Trauma Data Bank. Critically injured adults (ISS > 15) admitted to the intensive care unit (ICU) or operating room from the emergency department at Level I and II trauma centers were included. The outcome of interest was risk-adjusted mortality across quintiles of patient admission volume, modeled using mixed-effects generalized linear models. **Results:** In total, 802,824 patients were included, 321,209 and 481,615 in the early and late groups, respectively. In the early group, increased patient volume was associated with a decreased risk-adjusted odds of mortality. This association was not seen in the late group. The overall odds of mortality in the late group demonstrated decreased mortality over time (OR 0.84, *p* < 0.001). **Conclusions**: The annual volume of critically injured patients was associated with decreased odds of hospital mortality during 2007–2011, though this effect was no longer present in the 2017–2021 sample. The continued dissemination of the best practices is warranted to decrease mortality, regardless of the admission volume of critically injured patients.

## 1. Introduction

A presiding tenet of trauma center and trauma system development is that increasing trauma center volume improves outcome. It has been inculcated into the requirements of trauma center verification of the American College of Surgeons (ACS) that for a trauma center to be verified as a Level I trauma center, it must admit a minimum of 1200 patients annually who meet the National Trauma Data Standard inclusion criteria or annually admit 240 patients with an ISS > 15 [1]. The literature supporting such a mandate is ambiguous, with some studies supporting a volume/outcome relationship [2,3,4,5] while others not [6,7,8,9,10,11]. Many of the seminal studies on the trauma volume/outcome relationship are now more than ten years old [2,3,4,12]. Moreover, the patient selection criteria have varied widely among studies, with mixed findings reported.

Since the early 2000s, the standard of care offered at trauma centers and within trauma systems in the United States has continued to improve. At the national level, partnerships between military and civilian healthcare teams have facilitated a bidirectional flow of information, where military surgeons can maintain their skills during times of peace while allowing for a seamless transition of battlefield medical advances to the civilian sector [13]. Further, the number of Level I or II trauma centers has grown, resulting in an increase in the number of Americans with access to a Level I or II trauma center within an hour from 78% in 2013 to 91% in 2019 [14]. In those rural communities where access to a Level I/II trauma center is not readily available, the development of Level IV trauma centers has allowed those injured in austere environments to receive initial stabilization by treating life and limb threatening injuries before transfer to definitive care [15]. The creation of educational courses such as Advanced Surgical Skills for Exposure in Trauma (ASSET), Advanced Trauma Operative Management (ATOM), Disaster Management and Emergency Preparedness (DMEP), and Stop the Bleed has increased the accessibility and standardization of knowledge on caring for an injured patient. At the clinical level, the dissemination of the resuscitative endovascular balloon occlusion of the aorta (REBOA) [16], improvements in prehospital care [17], and the adoption of damage control resuscitation, which emphasizes limited crystalloid utilization with an emphasis on whole blood administration and the development of massive transfusion protocols [18,19], are just a few of the innovations that have contributed to the improved care of injured patients.

The conflicting results of the literature on the trauma volume/outcome relationship, and the advances in care within the last decade provide the rationale for this study. We sought to evaluate the effect of trauma center admission volume on risk-adjusted mortality among patients requiring admission to the intensive care unit (ICU) or operating room and compared the effects of admission volume between an early cohort (2007–2011) and late cohort (2017–2021). We hypothesized that in the early cohort, trauma volume would be associated with improved risk-adjusted survival, but in the late cohort, the trauma volume/mortality relationship would be mitigated due to trauma system maturation (improvement in the system level care provided) and an improvement in the overall ability of clinicians to care for the injured patient. This study was deemed exempt by the Institutional Review Board and Human Subjects Protection Program.

## 2. Methods

**Figure 1:**
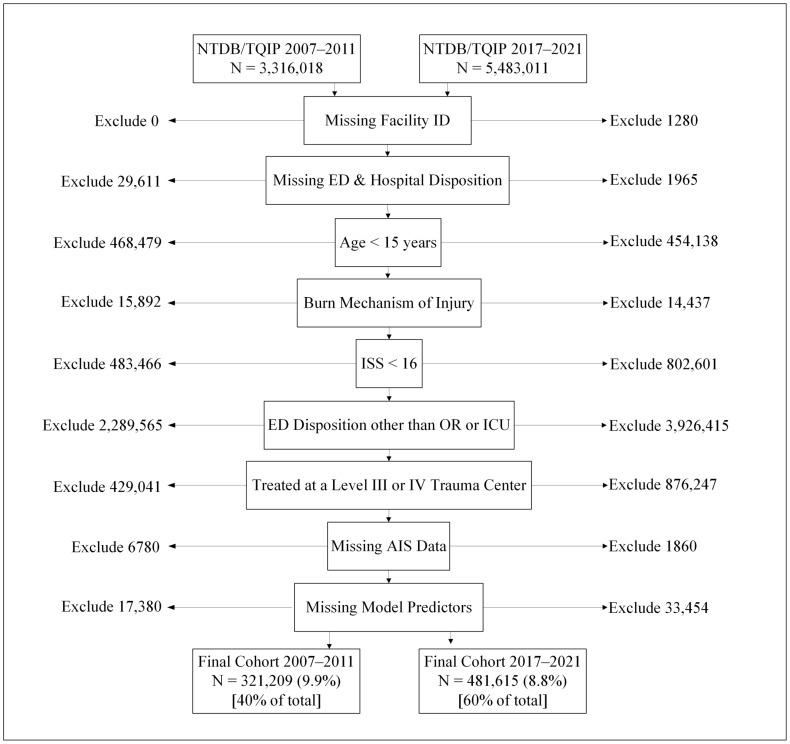
STROBE diagram of patient exclusion by time period.

We conducted a two-armed cohort study using Trauma Quality Program Participant Use Files (TQP PUF) from the American College of Surgeons’ National Trauma Data Bank (NTDB) data from two five-year periods, 2007–2011 (early) and 2017–2021 (late). We included all severely injured patients within a larger trauma center cohort at all levels of trauma volume using the ACS TQIP and National Trauma Data Bank databases. The time periods were selected because they were the most recent five years available in TQIP/NTDB and five years from a decade earlier for comparison. The patient sample examined in this study included critically injured (ISS > 15 and admitted to the ICU or operating room from the emergency department) adults (age ≥ 15 years-old) treated at a facility capable of providing definitive care (Level I or II trauma center). By only including such severely injured patients, we posit that the resources of a trauma center are tested, and the effect of care is manifested most readily. We excluded patients whose records lacked a facility identifier, those with missing emergency department or hospital disposition data, patients missing Abbreviated Injury Scale records, and patients with missing data for model predictor variables. Patients with a mechanism of injury classified as burns were also excluded from this study due to differing injury physiology. A flow diagram illustrating the exclusion criteria with the number of patients excluded for each criterion is shown in Figure 1. The exposure of interest was a quintile of the average number of critically injured patients admitted to each trauma center per year, while the outcome of interest was the risk-adjusted, odds ratio (OR) of mortality among the Level I and II trauma centers contributing data during the time periods described above.

To assess risk-adjusted hospital mortality, a mixed-effects generalized linear model (MEGLM) was fit by applying the process of purposeful selection as described by Hosmer, Lemeshow, and Sturdivant [20] with hospital identifier included as the random-effects parameter. The hierarchical model approach using hospital identifier as a random-effects parameter is consistent with other investigators’ approach to this topic [10,21,22,23]. Likelihood ratio tests and fit statistics were used to confirm the appropriateness of the inclusion of hospital as a random effect in both the early and late groups. The variables included in the model were age in years, male sex, the penetrating mechanism of injury, traumatic brain injury, and the Glasgow Coma Scale (GCS) motor score. The GCS motor score has been shown to be an excellent mortality predictor and has been promoted as a standalone neurologic component in mortality prediction models [24]. Additionally, the severity of anatomic injury was included in the model as the logit-transformed probability of mortality [*log(probability/1 − probability)*] as estimated by the trauma mortality prediction model (TMPM) developed and validated for the Abbreviated Injury Scale by Osler et al. [25,26]. Model discrimination was assessed using the area under the receiver operating characteristic curve (AUROC) [27].

Dichotomous and categorical data were presented as frequencies and proportions, then compared using the chi-squared statistic. Nonparametric continuous variables were presented as medians with the 25th and 75th percentile values and compared using the rank sum test. Estimates, including regression coefficients, for example, are presented as mean values with 95% CIs. Data manipulation and statistical analysis was conducted using Stata 17.0 MP, College Station, TX. *p*-values less than 0.05 indicated statistical significance. Note that the large sample size in this study produced very small *p*-values, even for clinically trivial effect sizes. We therefore emphasize effect sizes including the difference in means or proportions, odds ratios, and regression coefficients rather than *p*-values.

## 3. Results

In total, 802,824 patient records met the inclusion criteria for the study, including 321,209 (40.0%) from the early group (2007–2011) and 481,615 (60.0%) from the late group (2017–2021). Demographic, payer, comorbid conditions, transfer and trauma type, GCS, anatomic injury groups, injury severity measures, and ICU and hospital lengths of stay are compared between the early and late groups in Table 1. Notably, the early group was younger than the late group by a median difference of six years (44 years versus 50 years). The distribution of the Glasgow Coma Scale score (GCS) differed between patient groups. The median (25th percentile and 75th percentile) was 15 (6, 15) in the patients treated in the early time period, whereas the GCS was 14 (7, 15) in the late period, *p* < 0.001. The proportions of patients with a GCS score less than 15 differed between groups by 1.1% (49.4% in the early group and 50.5% in the late group). For measures of injury severity, the median ISS was 22 (25th and 75th percentiles: 17 and 29) in both groups, *p* < 0.001. The median (25th and 75th percentiles) probability of death as estimated by the TMPM was 5.3% (2.1%, 18.3%) in the late group, whereas it was 4.1% (2.0%, 10.3%) in the early group, *p* < 0.001. The median (25th and 75th percentiles) ICU length of stay was longer in the late group, 4 days (2 days and 8 days), versus the early group, 3 days (2 days and 9 days). The observed number of deaths was 51,282 (15.2%) of the early group and 82,780 (16.1%) of the late group, *p* < 0.001 (Table 1).

A total of 604 trauma centers cared for the patients in this study. Most centers were non-profit hospitals (86.1%) with fewer for-profit hospitals (13.3%) and only four identified as government hospitals (0.7%). Academic teaching centers accounted for one-third of the trauma centers (33.9%) in this study, whereas community hospitals represented the plurality (45.4%), and hospitals described as nonteaching constituted the remaining fifth (20.5%). ACS-verified trauma centers (n = 400) outnumbered state-designated centers (n = 121) nearly four to one. The largest group of hospitals, by level of verification or designation, was the ACS Level II centers, which numbered 230 (44.6%), followed by ACS Level I centers, n = 170 (32.6%). The state-designated centers were fewer, with the Level I centers accounting for 46 (8.8%) hospitals and Level II totaling 75 hospitals or 14.4% of all hospitals in the study. Nearly two-thirds of the hospitals contributed patient data to both time periods (61.9% or 374 centers) (Table 2).

Two MEGLMs were fit to model the effect of admission volume quintile on risk-adjusted mortality, conditioned upon injury severity (TMPM), age, sex, GCS motor score, TBI, and penetrating injury status. In the early group, greater volumes of critically injured patients were associated with decreased risk-adjusted odds of mortality. Relative to the lowest volume quintile, the third, fourth, and fifth quintiles were associated with lower odds of mortality (OR 0.83, 0.84, and 0.79, respectively, with *p* < 0.05 for all three). In contrast, in the late group, greater volumes of critically injured patients demonstrated no such association with the odds of mortality relative to the lowest quintile, with *p* > 0.05 for all quintiles (Table 3).

An additional MEGLM was fit to assess the effect of early versus late time period on mortality. The late period was associated with 16% decreased relative odds of mortality (OR 0.84, 95% CI 0.83–0.86, *p* < 0.001). Among the parameters included in the model for risk-adjustment, penetrating injury more than doubled the odds of mortality (OR 2.22, 95% CI 2.16–2.27, *p* < 0.001). Traumatic brain injury and GCS motor score had inverse associations with mortality (OR 0.72 and 0.65, respectively, *p* < 0.001 each). This suggests an overall improvement in the care of critically injured patients during the interval of this study (Table 4).

## 4. Discussion

Increasing volumes of critically injured patients was associated with improved mortality over a decade ago, but this effect was not evident in the more recent data, with consistent methodology applied to both time periods. Moreover, the relative risk-adjusted odds of mortality improved between time periods.

The results of this study are consistent with other retrospective studies. Within the early group (2007–2011), many studies found a volume/outcome relationship. Minei et al. (2014) [2], using data from 2006 to 2009 from the Resuscitation Outcome Consortium, a network of over 60 hospitals, looked at the trauma volume/outcome relationship in TBI patients and patients who arrived in shock. They found that for every increase of 500 trauma center admissions, there was a 7% decrease in 24 h and 28-day mortality in the TBI cohort, but there was no improvement in mortality for the shock cohort. Bell et al. (2015) [3], using a NTDB cohort of patients from 2008 to 2010, looked at whether average annual trauma volume affected complications, failure to rescue, or mortality. Regression analysis showed that a higher hospital volume was associated with decreased mortality but not complications or failure to rescue. Nathens et al. (2001) [4] looked at 31 Level I or II academic trauma centers participating in the University Health System Consortium Trauma Benchmarking Study, comparing low volume (<650 trauma admissions) to high volume (>650 trauma admissions). In this study, they found a very significant association between admission volume and outcomes, with improved survival (odds of death 0.02) for penetrating trauma patients admitted with shock for centers admitting more than 650 such patients per year but no benefit in patients without shock or patients with blunt trauma. Within the late group (2017–2021), select studies have demonstrated a mitigation of the volume/outcome relationship. Sewalt et al. (2020) [9], using the Trauma Audit and Research Network database from the United Kingdom, looked at all patients admitted to a major trauma center with an ISS > 15 from 2013 to 2016. Using a random-effects logistic regression model, the authors found no association between trauma admission volume and outcomes. Garner et al. (2024) [28] examined the relationship between volume and outcomes for patients with an ISS > 15 at major trauma services in New South Wales from 2010 to 2019. There was no relationship between admission volume and mortality, however, as admission volume increased with hospital length of stay (adjusted β; 0.024, 95% CI, 0.182–1.089, *p* = 0.006).

In contrast, within the early group, Glance et al. (2004) [6] studied the relationship between trauma center volume and outcome using the NTDB. Patients with an ISS > 15 from both blunt and penetrating trauma in 67 hospitals between 1994 and 1999 were considered, and 1 hospital was eliminated as a high outlier. Using logistic regression, they found no association between trauma center volume and outcome. Demetriades et al. (2005) [7] also looked at the NTDB and patients with an ISS > 15 with specific traumatic injuries. In this relatively narrow spectrum of very severe injuries, there was no effect of volume on outcome in either Level I or II trauma centers. London and Battistella (2003) [8] analyzed patients ≥18 years old admitted to a Level I or II trauma center using the Patient Discharge Data of the State of California from 1998 to 1999. They found hospital volume was not a significant predictor of mortality or hospital length of stay. Within the late group (2017–2021), Kojima et al. (2022) [22] looked at the volume/outcome relationship specifically within the geriatric population (≥65 years) with an ISS ≥ 16 during the years 2015–2019. The authors found that adjusted odds of in-hospital mortality decreased as the volume of geriatric patients or the proportion of geriatric to total trauma patients increased.

These studies primarily suffer from a small sample of trauma centers. The present study collected data from 604 Level I and II trauma centers compared to the 67 hospitals by Glance et al. (2004) [6], 248 trauma centers by Demetriades et al. (2005) [7], and 38 Level I and II trauma centers by London and Battistella (2003) [8]. Further, Demetriades et al. (2005) [7] focused on a specific subset of severely injured patients, and Kojima et al. (2022) [22] focused on geriatric patients, while London and Battistella (2003) focused on trauma centers in a specific geographic area, thus making the results of these studies unable to be generalized to the volume/outcome relationship across the United States in all severely injured trauma patients.

Over the last decade, the number of trauma centers has substantially increased, especially in developed urban and suburban areas, and as these trauma centers have matured, so have their outcomes [15]. The results of this study that mature trauma centers and systems have significantly lower mortality rates compared to their younger counterparts is well documented in the literature. Moore et al. (2018) [29] conducted a systematic review and meta-analysis on the specific components of trauma systems that allow them to reduce morbidity and mortality. They retained 41 studies for a qualitative synthesis and 19 for a meta-analysis. They found trauma system maturity was associated with a statistically significant reduction in patient mortality, although the quality of evidence for this conclusion was low. These results were further substantiated by Alharbi et al. (2021) [30] who conducted a systematic review and meta-analysis of articles published between 2000 and 2020. Fifty-two studies were included for quantitative analysis, and it was determined that treatment in an immature, pre-implementation trauma system was significantly associated with a greater mortality rate compared to treatment in a mature system by nearly 1.5-fold.

Advanced Trauma Life Support (ATLS) classes put on by the ACS have been around for nearly 50 years, allowing a standardized best practice for trauma care to be adopted across institutions. The standardized training of ATLS has been one of the many vehicles facilitating the excellent trauma care seen in the last decade, allowing trauma practitioners to bring improved care to their institutions, and may have resulted in better patient outcomes, regardless of admission volume. Further, in 2014, the ACS began to mandate trauma centers of every level to have a massive transfusion protocol in place, with failure to implement this protocol being considered a Type I Critical Deficiency [31]. While this study was not designed to measure the effect of massive transfusion protocols, this change in criteria occurred in the interval between the early and late time periods and may play a role in the difference in outcomes reported.

There are several threats to the validity of the results of this study. The retrospective nature of the study carries with it its own inherent limitations. The included patients were heterogenous with regard to the mechanism and anatomic complement of injuries in this analysis, which may have obscured any volume/outcome relationship present within specific injury groups. Groups were not stratified based on injury pattern, as the ACS admission volume mandate for Level I trauma centers does not specify volume requirements for specific injury patterns. There were also significant differences seen between the groups in the two time periods in terms of race, injury characteristics, and comorbidities. It is important to note that overall, the late group had a significantly higher predicted mortality, as referenced by the TMPM, yet had a lower observed/predicted mortality for all volume quintiles. Finally, the TQIP/NTDB database does not allow for the characterization of trauma center staffing patterns or physician experience, which may have accounted for the disparity seen between the two cohorts. Better staffed hospitals or more experienced physicians may have accounted for the decrease in risk-adjusted mortality in the late cohort.

## 5. Conclusions

This study contains the largest cohort of Level I and II trauma centers, examining the trauma volume/outcome relationship. Among critically injured patients in Level I and II trauma centers, risk-adjusted mortality is no longer a function of annual patient volume as it was over a decade ago. In this same interval, the overall risk-adjusted mortality improved. Continued efforts to disseminate the best practices and support the development of mature trauma systems are warranted to optimize patient outcomes, regardless of the trauma center admission volumes of critically injured patients.

## Figures and Tables

**Table 1:** Comparison of early and late groups, ISS > 15.

	Early Group 2007–2011	Late Group 2017–2021	*p*-Value
Total, n (%)	321,209 (40.0)	481,615 (60.0)	
Age, Years, Median (25%, 75%)	44 (26, 62)	50 (30, 68)	<0.001
Sex, Male, n (%)	230,102 (71.6)	342,657 (71.2)	<0.001
Race/Ethnicity, n (%)			<0.001
White	213,346 (66.4)	250,732 (52.6)	
Black/African American	42,447 (13.2)	66,342 (13.8)	
Asian	5064 (1.6)	9124 (1.9)	
American Indian/Alaska Native	2727 (0.9)	3698 (0.8)	
Native Hawaiian/Pacific Islander	431 (0.1)	957 (0.2)	
Hispanic/Latino	27,587 (8.6)	122,869 (25.5)	
Multiracial	10,166 (3.2)	812 (0.2)	
Other/Race Unknown	19,441 (6.1)	27,081 (5.6)	
Payer, n (%)			<0.001
Medicaid/Medicare	87,056 (31.1)	207,185 (43.0)	
Private Insurance	114,008 (40.8)	177,237 (36.8)	
Other Insurance	21,683 (7.8)	26,674 (5.5)	
Self-Pay	44,717 (16.0)	58,384 (12.1)	
Not Billed	2786 (1.0)	1590 (0.3)	
Missing/Unknown	9513 (3.4)	10,545 (2.2)	
Comorbid Conditions, n (%)			
Hypertension	66,660 (20.8)	145,171 (30.4)	<0.001
Smoking	23,342 (7.3)	102,330 (21.3)	<0.001
Diabetes Mellitus	27,635 (8.6)	61,094 (12.7)	<0.001
Alcoholism	33,307 (10.4)	42,219 (8.8)	<0.001
Dementia	18,480 (5.8)	16,705 (3.5)	<0.001
Functionally Dependent Health Status	1630 (0.5)	25,985 (5.4)	<0.001
Congestive Heart Failure	7295 (2.4)	15,845 (3.3)	<0.001
CVA/Stroke	6867 (2.1)	11,844 (2.5)	<0.001
DNR Status	1563 (0.5)	14,449 (3.0)	<0.001
Transfer from outside facility, n (%)	94,700 (29.5)	126,476 (26.3)	<0.001
Trauma Type, n (%)			<0.001
Blunt	276,211 (86.0)	408,491 (84.8)	
Penetrating	35,088 (10.9)	58,109 (12.1)	
Other/Unspecified/Unknown	9910 (3.1)	15,015 (3.1)	
GCS, Total, Median (25%, 75%)	15 (6, 15)	14 (7, 15)	<0.001
GCS <15, n (%)	158,588 (49.4)	243,202 (50.5)	<0.001
Injuries, n (%)			
Skull Fractures	81,256 (25.3)	116,831 (24.3)	<0.001
Traumatic Brain Injuries	200,775 (62.5)	297,852 (61.8)	<0.001
Facial Fractures	62,999 (19.6)	106,157 (22.0)	<0.001
Airway/Lung Injuries	82,433 (25.7)	121,675 (25.3)	<0.001
Cardiac Injuries	9171 (2.9)	8700 (1.8)	<0.001
Vascular Injuries	29,368 (9.1)	74,708 (15.5)	<0.001
Vertebral Fractures	91,932 (28.6)	153,054 (31.8)	<0.001
Neurological Injuries	32,082 (10.0)	59,587 (12.4)	<0.001
Hollow Viscus Injuries	22,961 (7.2)	43,802 (9.1)	<0.001
Intra-Abdominal Solid Organ Injuries	67,199 (20.9)	99,290 (20.6)	0.001
Pelvic Fractures	46,017 (14.3)	82,943 (17.2)	<0.001
Renal/Urinary Tract Injuries	23,779 (7.4)	39,333 (8.2)	<0.001
Extremity Fracture/Dislocation/Amputations	133,886 (41.7)	200,505 (41.6)	0.66
Chest Wall Fractures/Contusion/Lacerations	138,758 (43.2)	232,312 (48.2)	<0.001
Skin/Soft Tissue Injuries	180,466 (56.2)	336,050 (69.8)	<0.001
ISS, median (25%, 75%)	22 (17, 29)	22 (17, 29)	<0.001
TMPM Probability of Death,%, median (25% and 75%)	4.2% (2.0%, 10.3%)	5.3% (2.2%, 18.1)	<0.001
ICU Length of Stay, Days, Median (25% and 75%)	3 (2, 9)	4 (2, 8)	<0.001
Hospital Length of Stay, Days, Median (25%, 75%)	8 (4, 16)	9 (5, 16)	<0.001
Died, n (%)	48,277 (15.0)	76,791 (15.9)	<0.001

**Table 2:** Characteristics of trauma centers.

Hospital Type, n (%)	
Non-Profit	521 (86.3)
For Profit	78 (12.9)
Government	5 (0.8)
Teaching Status, n (%)	
Academic	201 (33.3)
Community	269 (44.5)
Nonteaching	130 (21.5)
Bed Size, n (%)	
200 or Less	135 (22.4)
201–400	221 (36.6)
401–600	114 (18.9)
More than 600	134 (22.2)
ACS Verification or State Designation Level, n (%)	
ACS Level I	161 (26.7)
ACS Level II	240 (39.7)
State Level I	68 (11.3)
State Level II	135 (22.4)
Included in Time Periods, n (%)	
2007–2011 Only	75 (12.4)
2017–2021 Only	155 (25.7)
Both Periods	374 (61.9)

**Table 3:** Risk-adjusted mortality by critical admissions per year modeled using MEGLM *.

Volume Quintiles	Critical Admissions/Year	Odds Ratio	95% Confidence Interval	*p*-Value
Early Group 1	1–134	Referent	--	--
2	135–241	0.89	0.75–1.06	0.19
3	242–419	0.83	0.71–0.98	0.03
4	424–662	0.84	0.71–0.98	0.03
5	665–2323	0.79	0.67–0.93	0.01
AUROC 0.889				
Late Group 1	12–219	Referent	--	
2	223–348	1.02	0.91–1.14	0.75
3	354–519	0.94	0.84–1.05	0.28
4	520–765	0.93	0.84–1.04	0.20
5	777–2678	0.91	0.82–1.02	0.10
AUROC 0.895				

* Conditioned upon injury severity (TMPM), age, sex, GCS motor score, TBI, and penetrating injury status.

**Table 4:** Effect of time period on risk-adjusted mortality using MEGLM.

	Odds Ratio	95% Confidence Interval	*p*-Value
Time Period, Late vs. Early	0.84	0.83–0.86	<0.001
TMPM	2.01	2.00–1.02	<0.001
Age, Years	1.04	1.04–1.04	<0.001
Sex, Male	1.08	1.07–1.10	<0.001
GCS Motor Score	0.65	0.64–0.65	<0.001
Traumatic Brain Injury	0.72	0.70–0.73	<0.001
Penetrating Injury Type	2.22	2.16–2.27	<0.001
AUROC = 0.891			

## Data Availability

The data used in this study is publically available through the American College of Surgeons Trauma Quality Improvement Program (TQIP) database at https://www.facs.org/quality-programs/trauma/quality/national-trauma-data-bank/datasets/.
